# Monthly Summary

**Published:** 1875-08

**Authors:** 


### MONTHLY SUMMARY.

---

*Billroth on the Repair of Tissues.*—The following statements are given by Billroth as embodying the most recent views on the reparative process:

When repair commences in muscular tissue that has been injured or inflamed, with accompanying loss of substance, the space will be filled by wandering cells; the broken ends of the muscular fibres will grow out a longer or shorter distance, ending in one or more minute points. Coincidently, many nuclei appear in the growing end of the fibre; whether these come from the old nucleus or are new formations is uncertain. It also appears as if there were a complete new formation of young muscle-fibres between the old ones. So far as we know, it is uncertain where the cells originate that produce these intercalated growths.

In each injured nerve-fibre, whether at the periphery or at the centre, the axis cylinder, and subsequently the medullary sheath, degenerate for a certain distance, while the whole neighborhood is filled with wandering cells. Then the axis cylinder grows forward inside the nerve-sheath, generally under the form of one or more cords.

The growing cords meet and may unite with one another. The axis cylinder of the nerves is to be regarded as a long, protoplasmic process of a ganglion cell; if it be a cut in any place, it grows again simply by a lengthening or protusion of its substance. When the nerve fibres in the tadpole's tail bud out, the future nuclei of the nerve-sheaths form spontaneously in the nerve-bud, without any participation of the existing nuclei of the nerve-sheath.

Most of the studies on the regeneration of epithelium have been done on the cornea, and the following facts have been ascertained: when repair commences, the epithelium cells that lie on the margins of the lost tissue put out buds, which become broader, and then separate to form independent cells. Some believe that the nuclei are obtained by a division of the nucleus in the budding-cell, others do not.

When repair takes place in vessels, pointed buds appear at at the cut ends of the vessels a few days after the injury; some are short and some are long; in union by first intention they stretch over as far as the opposite side of the wound; when granulations form, they arch over and form loops.

We know nothing definitely about the formation of connective tissue, and the prevailing idea is that it can only be regenerated through the medium of young cells, but it is uncertain where these cells originate. Billroth favors an old view published by him previous to 1869, that the tissue is formed from the wandering cells, which are themselves derived from the connective tissue by budding. This is very similar to the view of Rokitansky, that in inflammation the tissue softens and then buds or forms branching excrescences, and indeed it is in accordance with what is seen in serous membranes, for vegetations often form in them in chronic inflammations; it is also known that certain sarcomatous tumors grow in a similar way.—*Wien. Med. Woch.—Med. Record.*

---

*Camphorated Chloral in Neuralgia.*—Dr. Lenox Browne, (*British Medical Journal*, March 7th,) employs as a valuable topical application in neuralgia a mixture of equal parts of chloral-hydrate and gum-camphor. In every case he has found great and often instantaneous relief to follow its application. "Its success," he writes, "does not appear to be at all dependent on the nerve affected, it being equally efficacious in neuralgia of the sciatic as of the trigeminus. I have found it of the greatest service in neuralgia of the larynx, and in relieving spasmodic cough of a nervous or hysterical character. It is only necessary to paint the mixture lightly over the painful part, and allow it to dry. It never blisters, though it may occasion a tingling sensation of the skin. My friend, Mr. George Wallis, allows me to say that he has found it of great service as a remedy which patients can apply themselves for the relief of toothache; and to its success in this respect I can also personally testify. In the original article, the compound was recommended for arresting the progress of incipient boils and carbuncles. I have no experience of its value for this purpose."—*Druggists' Circular*.

*Consanguineous Marriages.*—This was the subject of a recent paper by Mr. Darwin, son of the eminent naturalist. He stated that the most thorough investigation ever made was contained in some papers *On Blood Relationship in Marriage*, published by Dr. Arthur Mitchell, a Deputy Commissioner for Lunacy in Scotland. This gentleman's inquiries were confined chiefly to Scotland, in which country the proportion of first-cousin marriages is larger than in any other portion of the United Kingdom; and the conclusion he arrived at was that, under favorable conditions of life, the apparent ill-effects were frequently almost *nil*, while if the children were ill-fed, badly housed and clothed, the evil might become very marked. This, said the lecturer, was in striking accordance with some unpublished experiments of his father's, "On the In-and-in Breeding of Plants," in which he found that in-bred plants, when allowed space enough and good soil, would frequently show little or no deterioration; but when placed in competition with another plant, would frequently become stunted or altogether perish.—*Med. & Surg. Reporter.*

---

*The Teeth as Sexual Organs.*—Dr. A. H. Thompson, in the *Dental Cosmos*, says, "As prehensory organs, the value of the teeth is great to many of the lower forms, for battle, as "secondary sexual organs," and the prehension of food, but in the higher animals the teeth have been superseded by the evolution of prehensile limbs, and the consequent suppression of this function in the teeth. In man we find the prehensory teeth suppressed to mere rudiments of former formidable and useful weapons, being reduced to the merest purpose for the prehension of food, and are utterly aborted as secondary sexual organs. This is a fact that cannot be mistaken, and ls full of significance."

---

*Medical and Dental Graduates.*—The Medical Department of Nashville and Vanderbilt Universities has just graduated fifty-eight students. The Medical Colleges of the University of Wooster, thirty; the School of Medicine in the University of Maryland at Baltimore, fifty; the College of Physicians and Surgeons at Baltimore, thirty-nine; the Baltimore College of Dental Surgery, in the same city, seventeen; Yale, seven; the College of Physicians and Surgeons, Med. Dep. of the Syracuse University, eleven, one of whom was a female; the N. Y. College of Homœopathy, thirty-nine; the N. Y. College of Dentistry, sixteen.

*Action of Mercury Upon the Blood.*—Dr. Wilbouchewitch, of Moscow, (*Archiv. de Physiologie,*) has made a practical application of the ingenious apparatus devised by Mahassez for counting the blood corpuscles. This apparatus briefly consists of a fine capillary glass tube of definite capacity, so small as to bear a microscopic examination with an object glass capable of defining the blood corpuscles. The blood is diluted with artificial serum, placed in the tube, the corpuscles counted. By a simple calculation it is easy to ascertain the number of corpuscles in a given amount of blood.

He found that in the healthy subject the number of blood discs averaged 4,600,000 to the cubic milimetre. In different individuals it varied from four to six millions, but in the same subject the number remained pretty constant.

In syphilitic subjects examined before the beginning of specific treatment, the author found a progressive decrease of blood corpuscles amounting generally to more than 200,000 daily. As soon as mercurial treatment was instituted (of one and two-thirds grains of proto-iodide was begun) the blood corpuscles began to increase in number at the daily rate of 120,000 daily. This increase continued during two or three weeks only. Then the number began to again diminish, so that in a fortnight the number ofdiscs was about the same as at the beginning of treatment. If now the treatment was suspended, the destruction of corpuscles ceased, and in about a week the blood began to grow richer at a daily rate of about 90,000. As a general rule the white corpuscles underwent variations inverse to those of the red corpuscles.

These experiments teach that the administration in syphilis should be intermittent in order that the drug shall not itself act as a destructive agent upon the blood corpuscles—*Boston Medical Journal.*

---

*Epistaxis Arrested by Compressing the Facial Arteries.*—Dr. Beverly Robinson succeeded in arresting a hemorrhage from the nose, after the failure of other methods, including the plug of India-rubber bag, by constant pressure applied to the facial arteries. The pressure was made on the superior maxillary bones, just before the arteries reach the alæ nasi. Two small pads of lint were sewed to a tape at the proper distance apart, and the ends of the tape were passed around the head above the ears, and tied securely behind. The bleeding immediately ceased, and returned only when the pressure was removed for a short time. In a few days the cure was complete and permanent. Iron and tonics were also employed. If the plan should prove generally applicable and efficacious, Dr. Robinson will receive a liberal measure of gratitude and honor.—*New York Medical Record.*

*New Method of Arresting Hemorrhage.*—Dr. Webber, in a clinical lecture, describes a new method of arresting arterial hemorrhage. The principle on which his method is based, is to increase the circular contracting force of the arteries. If powerful enough, it is evident that this force would be as efficient as in the smaller arteries. To effect his object, he turns the end of the vessel inside out, as we would turn up the cuff of a sleeve. Thus he doubles or trebles the amount of contractile force. In executing his notion, he obtained an instrument by which he could fix the vessel at the time of turning over its coats. In experiments on dogs, he obtained satisfactory results, and found that he could prevent the vessel from becoming straight again, after the reflection had been made, by simply notching its extremity at each end of a transverse diameter. The notch was made through the two walls—the main and the reflected, and commencing at the point of the reflection, extended about two lines in a longitudinal direction. If this proves insucffiient to retain the reflection *in situ*, he introduces immediately behind the point of reflection a delicate little peg, made of the end of a number twelve English sewing needle. Should this fail to arrest bleeding at once, he applies digital compression for a short time to the end of the artery. He has successfully tried this plan on the anterior and posterior tibial, the brachial and femoral arteries.—*Medical Record.*

---

*Solubility of Salicylic Acid.*—Salicylic acid is very sparingly soluble in cold water, requiring at least five hundred times its weight of that fluid to hold it in permanent solution. The addition of certain alkaline salts increases its apparent solubility. Among these sodium phosphate has been especially recommended. It seems probable that an acid phosphate and a salicylate of sodium are formed, as the solubility of the sodium phosphate itself is also greatly increased. If this is the true explanation, it is probable that the antiseptic powers of the salicylic acid are partially lost in the solution made of the acid by this salt. Ammonium phosphate has also been employed, but is probably liable to the same objection. Sulphite of sodium is now recommended as yielding a solution possessing antiseptic powers of a high order. One part of salicylic acid, two of sodium sulphite, and fifty of water, furnish a solution free from irritating properties, and especially applicable to the treatment of zymotic diseases.

Glycerine warmed will dissolve one-fiftieth of its weight of salicylic acid, and the solution may then be diluted with water to any desirable extent.—*Detroit Review of Med. and Phar.*

*Structure of Tumors as Related to their Character.*—Dr. W. S. Savory directs attention to the structure of tumors, as indicating their character and clinical history.

Thus, tissues which are simplest in structure, and mode of development, are most apt to form the substance of tumors, as, for example, connective tissue. The most elaborate tissue, in these respects, striated muscle, is very rarely, if ever, found as a distinct morbid growth, and it is comparatively rare to find even unstriped muscle as a separate mass in the substance of tumors.

In the elementary structures of tumors there is an inverse ratio between the tendency to progressive and destructive metamorphosis. The less the structures of which a tumor is composed tend to change from their primary or embryonic form, the more abundantly will they multiply, so that those tumors whose structures retain most nearly their primary form, are the most malignant. And as the structures of a tumor are capable of transformation, so do they lose their power of repetition, so that those tumors that consist most completely of fully formed tissue are the most innocent.—*British Medical Journal.*

---

*Narcotizing Horses.*—The *Gaz. Med. de Bordeaux* says that an eminent veterinary surgeon has informed the Medical and Surgical Society of that city that the coachmen of certain families had been for some time in the habit of administering chloral to the horses in their charge, so as to make them easier to ride or drive. It appears that the drug acted like a charm, for horses which had previously been so spirited as to give much trouble to their driver, became as quiet as lambs after a few days of this hyposthenic treatment. The change naturally attracted the attention of the owners of the animals, and they sent for the veterinary surgeon to ascertain the cause of the sudden gentleness. That functionary noticed a certain tendency to sleep in the animals, but scarcely knew to what to refer this unusual condition, when, in one of his visits, he chanced to find a bottle half full of chloral. When the coachman was questioned regarding the use he made of the drug, he confessed, after much hesitation, that, following the advice of a brother "whip," he gave the horses a dose of chloral every morning to make them go quietly—and further, that many of the fraternity in Bordeaux followed the same plan.—*Medical Record.*

---

*Women-Doctors in Russia.*—The Russian journals announce that the first bevy of women, [fourteen in number,] trained as assistant-surgeons, under the charge of the Ladies' Committee of St. Petersburgh, will complete their three years of prescribed studies this month, [December,] and be prepared for service.—*Lancet.*

*Relation of Alcohol to Animal Heat and Muscular Power.*—Dr. Richardson, in a series of lectures, adduces a series of facts, from which the following are logical deductions:

Alcohol is disposed of in the living body by other processes than simple direct elimination. But alcohol is not a builder of any tissue, even of fatty tissue. During every stage of its administration, except the first, alcohol reduces animal temperature. Thus it is not burned after the manner of food that supports combustion. It is decomposed into secondary products by oxidation, at the expense of the oxygen, which ought to be applied to the natural heating of the body. Further, as animal heat is reduced, so is the excitability of muscle, until at last, as narcotism is developed by the spirit, the muscular power is brought down to the extreme of prostration.—*London Lancet.*

---

*Snow as a Hœmostatic.*—Dr. Hane (*Centralblatt far Chirurgie* No. 38, 1874) recommends very highly snow as a means of preventing hemorrhage in those operations in which Esmarch's method cannot be used. A handful of dry snow pressed upon the bleeding wound he found in a case of tracheotomy to act most favorably, soaking up the blood like a sponge and arresting the bleeding.

---

*Warts.*—Dr. Guttceit recommends rubbing warts, night and morning, with a moistened piece of muriate of ammonia. They soften and dwindle away, leaving no such white mark as follows their dispersion with lunar caustic.—*Druggists' Circular.*

---

*Nitrous Oxide in Tuberculosis.*—Dr. C. W. Larison reports a case of miliary tuberculosis, in which, after the failure of all ordinary remedies, nitrous oxide was used with most desirable results. Improvement was very slight at first, but steadily increased until complete recovery resulted. Cutaneous circulation assumed its vigor, muscles performed their functions with greater precision, the pulse became stronger and less frequent, appetite became voracious, weight increased, etc. The amount of gas taken was about five gallons a day at intervals.—*Cinn. Med. News.*

---

*A Convenient Local Anodyne.*—Take of elastic collodion, one ounce; hydro-chloroform of morphia, fifteen grains. Dissolve the morphine salt in the collodion. Spread, with a camel-hair brush, some of this solutiou on the painful part, and place some oiled silk over the spot. The effect is stated to be most satisfactory.—*Med. & Surg. Reporter.*

